# Depression in Multiple Sclerosis: A Clinical Primer for Psychiatrists

**DOI:** 10.1177/07067437251396765

**Published:** 2025-11-18

**Authors:** David E. Freedman, Anthony Feinstein

**Affiliations:** 1 Department of Psychiatry, 71545 Sunnybrook Health Sciences Centre, Toronto, Canada; 2 Department of Psychiatry, Temerty Faculty of Medicine, 7938 University of Toronto, Toronto, Canada; 3 BARLO MS Centre, 10071 St. Michael's Hospital, Toronto, Canada

**Keywords:** depressive disorders, multiple sclerosis, neuropsychiatry

## Introduction

Multiple sclerosis (MS) is an immune-mediated demyelinating neurological disease that affects approximately 1 in 350 Canadians.^1^ Depression is common in people with MS (pwMS) but is often under-recognized and under-treated.^2^ It is thus essential for mental health clinicians to be confident in diagnosing and managing depression in the context of MS. Tailored for the practicing psychiatrist, this article provides a clinical primer about depression in MS, focused on its prevalence and impact, assessment, biopsychosocial correlates and management.

## Prevalence of Depression in Multiple Sclerosis

Depression is 3 times more common in pwMS than in the general population.^2^ The prevalence of depression does not appear to differ according to age or sex.^2,3^ This latter point merits emphasis, considering its contrast with well-described sex differences in depression in the general population. Reasons for this are unclear.

Depression contributes to reduced quality of life, medication non-adherence, unemployment, and elevated mortality in those with MS.^2^ Furthermore, pwMS are at elevated risk of suicide compared to the general population.^2^ These findings emphasize depression's impact on the lives of pwMS and the value of neuropsychiatric care.

## Correlates of Depression in Multiple Sclerosis

MS depression is associated with increased lesion volume within a “depression network,” consisting of the prefrontal, retrosplenial, and medial temporal cortices and the ventral tegmental area.^4^ Other biological correlates include gray matter atrophy and immunological abnormalities such as elevated pro-inflammatory cytokines.^2^ These findings indicate that depressive symptoms are intertwined with neuro-inflammatory and neurodegenerative factors in MS.

Depression in MS is also associated with personality traits (e.g., elevated neuroticism) and other symptoms, including sexual dysfunction, fatigue, pain, and anxiety.^2^ A problem-focused coping style, compared to emotion- or avoidance-oriented coping, has emerged as a protective factor against depressive symptoms in MS.^2^

## Link Between Depression and Cognitive Dysfunction in Multiple Sclerosis

Cognitive impairment affects 30% to 90% of pwMS and contributes to reduced quality of life, difficulties with completing activities of daily living, and unemployment.^2^ Commonly affected domains include processing speed, memory, learning, and executive function. A recent study demonstrated a link between depression, particularly with comorbid anxiety, and cognitive dysfunction in MS.^5^ This association remained even after controlling for relevant demographic and disease-related variables. Anxiety alone was not associated with cognition in MS.^5^ These data clarify equivocal evidence to date on this topic. Future studies are necessary to answer the vital question of whether successful treatment of depression improves cognition in MS.

## Assessment of Depression in Multiple Sclerosis

Depressive symptomatology in MS is characterized by increased irritability and a chronic course of symptoms.^2^ As part of the clinical assessment, one must consider comorbid symptoms (such as anxiety), the timeline of depressive symptoms in relation to the MS clinical course, and a cognitive assessment. Although cognitive screening is recommended for all pwMS, a comprehensive cognitive evaluation is recommended if there is a positive screen or evidence of cognitive decline.^6^ The Montral Cognitive Assessment is not validated in pwMS. The Symbol Digit Modalities Test (SDMT) and the Minimal Assessment of Cognitive Function in MS battery are recommended cognitive screening (for pwMS over age 8) and comprehensive assessments, respectively.^6^ For practicing psychiatrists, the SDMT takes 5 minutes to administer and a positive screen indicates the need to refer for comprehensive cognitive evaluation.^6^ Yet, access to neurocognitive testing is often limited. One must also screen for bipolar disorder as part of the depression assessment more so given the elevated risk of this condition in pwMS.^2^ There is insufficient evidence to recommend measurement-based care (MBC). Nevertheless, given expanding integration of MBC into general psychiatric care, validated depression symptom rating scales in pwMS include the Patient Health Questionnaire, the Beck Depression Inventory-II, and the Hospital Anxiety and Depression Scale.^2^

## Management of Depression in Multiple Sclerosis

A biopsychosocial understanding of depression in MS begets a multi-faceted approach to management. Cognitive-behavioural therapy (CBT) and mindfulness-based interventions are effective in reducing depressive symptoms in pwMS.^2^ CBT may also reduce pain, improve sleep, and foster social connections.^7^ A systematic review of 28 qualitative studies of CBT involving clinicians and pwMS suggests potential facilitators of therapy effectiveness.^7^ These factors include tailoring therapy to participant needs, financial and online accessibility, involving care partners, and incorporating opportunities for social connections between therapy participants. Although greater physical disability and prolonged disease duration may be independently linked to reduced odds of receiving psychotherapy in MS,^8^ reasons for this are unclear, highlighting another potential avenue for future study.

Few studies have evaluated whether antidepressant medications are effective in pwMS, and mixed findings in small samples limit the clinical application of these data.^9^ Nonetheless, antidepressants are widely prescribed to pwMS.^2^ In this context, antidepressant selection may be influenced by guidelines from the general population and co-occurring symptoms.^9^ Examples of commonly prescribed antidepressants include bupropion (e.g., in pwMS with sexual dysfunction and fatigue), duloxetine (e.g., in those with chronic pain), and sertraline (e.g., in pwMS who need small dose increments due to difficulties with tolerability). Other factors to consider include cardiovascular comorbidities, the risk of falls, drug-drug interactions, and adverse effects of polypharmacy.^2^ Caution is warranted with using second-generation antipsychotic medications to address depression considering limited evidence in pwMS, and risks of exacerbating cardiovascular comorbidities, fatigue, and extrapyramidal side effects.

## Conclusion

Depression is a manageable contributor to increased morbidity and mortality in pwMS. This primer highlights recent advances in knowledge on the subject. Yet, as our primer makes clear, many questions remain unanswered. What is more certain is that the diagnosis should not be missed or assumed to be an inevitable consequence of this illness. This oversight comes with a failure to treat, doing pwMS a grave disservice.

## Clinical Pearls

- Correlates of depression in MS include “depression network” lesions, gray matter atrophy, elevated pro-inflammatory cytokines, heightened neuroticism, and emotion- or avoidance-based coping styles.
- In people with MS, depression is independently linked to cognitive dysfunction.
- Cognitive-behavioural therapy and mindfulness-based interventions reduce depressive symptoms in people with MS; however, there is limited MS-specific evidence to guide the selection of pharmacotherapy.
